# Egg maturity assessment prior to ICSI prevents premature fertilization of late-maturing oocytes

**DOI:** 10.1007/s10815-018-1393-0

**Published:** 2019-01-12

**Authors:** Zuzana Holubcová, Drahomíra Kyjovská, Martina Martonová, Darja Páralová, Tereza Klenková, Pavel Otevřel, Radka Štěpánová, Soňa Kloudová, Aleš Hampl

**Affiliations:** 1 Reprofit International, Clinic of Reproductive Medicine, Hlinky 122, 603 00 Brno, Czech Republic; 2 Faculty of Medicine, Department of Histology and Embryology, Masaryk University, Kamenice 3, 625 00 Brno, Czech Republic; 3 Faculty of Medicine, Department of Pharmacology, Masaryk University, Kamenice 5, 625 00 Brno, Czech Republic

**Keywords:** Timing of ICSI, Oocyte maturation, Polarized light microscopy, Meiotic spindle, Immature oocytes

## Abstract

**Propose:**

The presence of metaphase II (MII) spindle together with the polar body (PB) indicates completion of oocyte maturation. This study was designed to explore if spindle imaging can be used to optimize timing of intracytoplasmic sperm injection (ICSI).

**Methods:**

The study involved 916 oocytes from 234 conventionally stimulated ICSI cycles with an unexpectedly poor ovarian response. All PB-displaying oocytes were subjected to polarized light microscopy (PLM) prior to ICSI. When MII spindle was absent in the majority of oocytes, ICSI was postponed and performed after additional spindle imaging. Fertilization, embryo development, and clinical outcome were evaluated with respect to the observed spindle pattern.

**Results:**

The visible spindle was absent in 32.64% of PB-displaying oocytes. The late-maturing oocytes extruding PB in vitro were less likely to exhibit a spindle signal than in vivo matured MII oocytes (38.86% vs. 89.84%). When fertilization was postponed, 59.39% of initially spindle-negative oocytes developed detectable MII spindle. Spindled eggs had significantly higher developmental potential, and the presence of the spindle has been identified as an independent measure for predicting the formation of the blastocyst. Embryos derived from spindle-positive oocytes also showed a higher chance to implant and develop to term. Notably, 11 children were conceived by finely timed fertilization of late-maturing oocytes which are normally discarded.

**Conclusions:**

The study confirms the prognostic value of spindle imaging and demonstrates that immature oocytes can be clinically utilized and give rise to live births when the timing of ICSI is adjusted to their developmental stage.

**Electronic supplementary material:**

The online version of this article (10.1007/s10815-018-1393-0) contains supplementary material, which is available to authorized users.

## Introduction

Oocyte maturity is an essential prerequisite for its successful fertilization and the viability of the resulting embryo. In clinical practice, it is customary to assume that each oocyte displaying a polar body (PB) is a mature metaphase II (MII) oocyte. However, live imaging of microtubule dynamics during human oocyte maturation revealed that PB becomes visible a couple of hours before the bipolar MII spindle is assembled [1]. Due to this asynchrony, MII-arrested eggs and telophase I oocytes cannot be distinguished by their appearance under a conventional microscope. If the sperm is injected in an unphysiological time, the developmental potential of late-maturing oocytes will be diminished [2–5].

Avoiding untimely intracytoplasmic sperm injection (ICSI) is particularly important in the population of poor/slow responders with a low number of eggs available for fertilization. Only PB-displaying oocytes are subjected to ICSI while germinal vesicle and metaphase I (MI) oocytes are typically discarded. However, immature oocytes, which spontaneously extruded PB in vitro, have been successfully used in fertility treatment [6–10]. Cumulative evidence suggests that the developmental potential of late-maturing oocytes could be better exploited if the timing of ICSI is adjusted to their developmental stage [2–5, 11–14].

The advent of computer-assisted polarized light microscopy (PLM) has enabled real-time assessment of egg maturity prior to ICSI. Detectable birefringence is generated by the interaction of a polarized light with the highly ordered microtubular mass building up the bipolar spindle. Due to its non-invasiveness, this technique could be used in clinical settings to monitor the dynamics of division apparatus in a living state [3, 15, 16]. Numerous studies have explored the possibility of predicting oocyte developmental ability by the evaluation of presence [11, 17–20], morphology [21–23], birefringence intensity [24–26], and positioning of the MII spindle with respect to the PB [17, 27–29]. The majority of these studies reported that the absence of the spindle compromises the ability of the oocyte to be fertilized, undergo preimplantation development, and give rise to full-term pregnancy [11, 17–19, 21–24]. Importantly, an experiment performed by Montag and colleagues demonstrated that the absence of the spindle birefringence might be only temporary, corresponding to the physiological transition from MI to MII stage [2]. In light of these findings, it is assumed that at least some late-maturing oocytes would benefit from the postponement of ICSI instead of being subjected to immediate sperm injection.

The primary objective of this study was to determine if PLM could be used as a tool to optimize the timing of ICSI with respect to the maturational stage of the oocyte. We sought to test the hypothesis that postponing ICSI of late-maturing oocytes might be beneficial because it provides more time for the emergence of the MII spindle, which is known to be associated with better clinical results.

## Materials and methods

### Study population and ethical approval

This prospective case series study was carried out in a single private IVF center, Reprofit International (Brno, The Czech Republic). It was comprised of a total of 916 oocytes from 229 IVF patients (aged 25–48 years, average 36.96 ± 4.28 years) undergoing 234 ICSI cycles between May 2016 and May 2018. Study participants were included only if the number of PB-displaying oocytes retrieved in stimulated cycles was lower than 6 and if there was a large proportion of immature MI oocytes which extruded PB in vitro within 3–4 h after retrieval (MI/MII oocytes). Severe male infertility factor was present in 14.96% of analyzed cases. In 15.38% of cycles, only immature oocytes, not MII oocytes, were collected. All included cycles were assigned to ICSI and extended embryo culture. Apart from the study population, 404 MII oocytes from 50 egg donors (aged 20–32 years, average 26.42 ± 2.99 years, no previous history of infertility) were used as a positive control of the spindle imaging procedure. Written informed consent was obtained from all participants. The study design was approved by the institutional Ethics Committee.

### Ovarian stimulation and oocyte retrieval

Ovarian stimulation was induced with either recombinant follicle-stimulating hormone (Gonal-f, Merck Serono, Switzerland; Puregon, MSD, USA) or highly purified human menopausal gonadotropin (Menopur, Ferring, Switzerland). Pituitary suppression was achieved by administration of the gonadotropin-releasing hormone antagonist (Cetrodite, Merck Serono, Switzerland). Ovulation was triggered with human chorionic gonadotropin (hCG) (Ovitrelle, Merck, Switzerland). Oocyte pick-up (OPU) was scheduled 35–36 h after hCG injection. Cumulus-oocyte complexes (COCs) were collected in a MOPS/HEPES-buffered medium (MHM, Irvine Scientific, USA). Oocyte denudation was undertaken immediately after retrieval. COCs were briefly exposed to hyaluronidase (#90101, Irvine Scientific, USA) and cumulus-corona cells were mechanically removed by gentle pipetting. Developmental status of the oocyte was assessed according to the presence or absence of the first PB. MI and MII oocytes were incubated separately in Continuous Single Culture (CSC) medium (#90164, Irvine Scientific, USA) for an additional 3–4 h at 37 °C in a humidified atmosphere of 5% O_2_ and 6% CO_2_. Oocytes, which extruded PB in vitro during the pre-incubation period, are hereby referred as “MI/MII oocytes” while term “MII oocytes” is restricted to the oocytes displaying PB at the time of denudation/retrieval.

### Polarized light microscopy

**Fig. 1:**
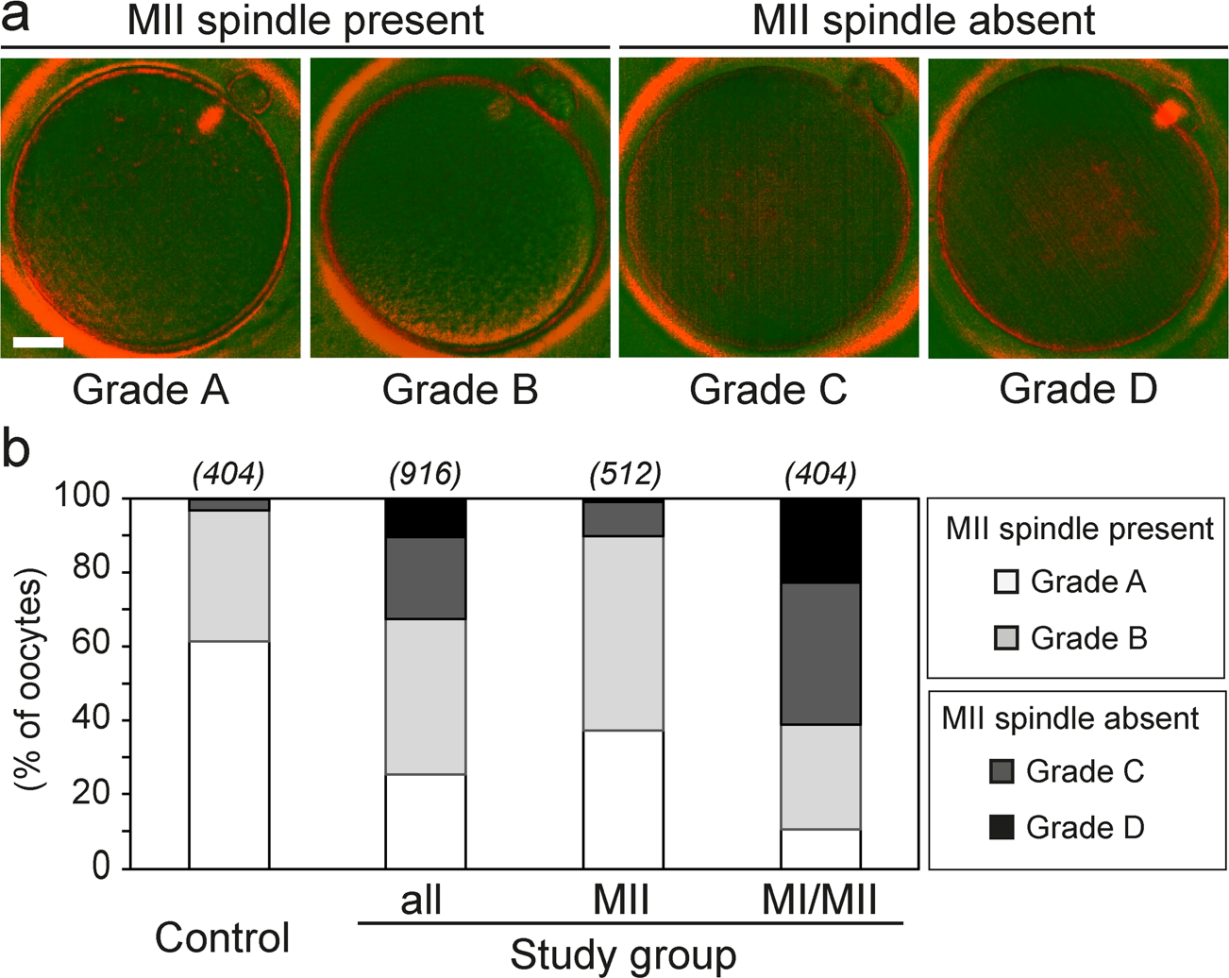
Categories and incidence of oocyte grades. (a) Representative examples of different oocyte grades based on PLM-detected pattern. Scale bar; 20 μm. (b) The incidence of oocyte grades (A–D) in the control group of oocytes (egg donors) and study group (poor/slow responders). Distribution of oocyte grades in subpopulations of MII and MI/MII oocytes is shown along with overall distribution. The total number of oocytes in each group is shown in brackets

All PB-displaying oocytes (MII and MI/MII) were subjected to maturity assessment prior to ICSI that was scheduled 39–40 h after hCG trigger (3–4 h after denudation). The oocytes were placed individually into numbered 5-μl droplets of a prewarmed HEPES/MOPS-buffered medium covered with equilibrated mineral oil (#9305, Irvine Scientific, USA) on glass-bottomed dishes (WPI Fluorodish FD 5040 or WillCo GWST-5040). PLM examination was performed on a Nikon Eclipse TE 2000-U microscope (Tokyo, Japan) equipped with OCTAX polarAIDE™ (MTG, Germany). Imaging software OCTAX Eyeware ™ (MTG, Germany) combined bright field (green) and birefringence (red) visions of individual oocytes while they were gently rotated around each axis to achieve spindle alignment with the path of polarized light. All micromanipulation procedures were carried out in a temperature-controlled environment maintaining 37 ± 0.5 °C in the culture droplet. PLM-examined oocytes were categorized based on the pattern of detected birefringence (Fig. 1a). Grade A oocytes featured a bipolar barrel-shaped spindle with clearly delineated boundaries and even distribution of birefringence, while grade B oocytes displayed dysmorphic, apolar, and translucent spindles with irregular boundaries and uneven distribution of the signal. Oocytes with no visible spindle birefringence were classified as a grade C. Anaphase I/telophase I oocytes, showing a microtubular bridge (a connective strand between first PB and the oocyte) instead of MII spindle, were marked as grade D oocytes. If the majority of oocytes in the treatment cycle were found to lack the MII spindle (grades C and D), sperm injection was postponed and performed 1–5 h (on average 2 h) later after additional PLM examination. The spindle imaging procedure was video recorded and the grading of each oocyte was performed by two independent evaluators.

### ICSI, embryo culture, and embryo transfer

All eggs underwent ICSI immediately after PLM (re-)assessment. They were placed into 5-μl droplets of a prewarmed HEPES/MOPS-buffered medium, covered by mineral oil on plastic microinjection dishes (Nunc™ IVF Petri Dishes, #150270, Thermo Fisher Scientific, Waltham, USA). ICSI was routinely performed according to the standard protocol using ICSI/holding micropipettes (#002-5-30/#001-120-30, Microtech IVF, The Czech Republic), polyvinylpyrrolidone (#90121, Irvine Scientific, USA), and Eppendorf (Hamburg, Germany) micromanipulation system equipped with thermoplate (TokaiHit, Japan). After ICSI, injected oocytes were placed into 30-μl droplets of CO_2_-dependent CSC medium covered with mineral oil in micro-droplet culture dishes (#16003, Vitrolife, Sweden) and individually cultured at 37 °C in a humidified atmosphere of 5% O_2_ and 6% CO_2_. Embryo development was followed until day 5 or day 6 when good-looking blastocysts were either transferred in a fresh cycle or cryopreserved (Rapid-i, #10119, Vitrolife, Sweden). Blastocysts were chosen for transfer based on morphological criteria by clinical embryologists who were blinded to the PLM assessment results.

### Study endpoints and data analysis

Primary outcomes were spindle visualization during PLM (re-)examination, fertilization, embryo blastulation, and utilization. ICSI outcome was analyzed with respect to the oocyte grades (A–D). Besides, developmental potential of oocytes with (grade A or B) and without (grades C and D) the MII spindle was compared. Fertilization was defined as the presence of two pronuclei 16–20 h post-ICSI. Blastulation (BR) and utilization rates (UR) were calculated as the total number of blastocysts/utilized embryos (i.e., transferred in the fresh cycle or cryopreserved) divided by the number of injected oocytes. Secondary outcome measurements included biochemical pregnancy rate (PR—number of positive hCG tests on day 10 post embryo transfer per number of embryo transfers) and clinical pregnancy rate (CPR—number of pregnancies with detected fetal heartbeat after 12 weeks of gestation per number of embryo transfers). Information about treatment outcomes, including abortions (loss of clinical pregnancies) and live births, was extracted from the electronic registration system.

### Statistical methodology

Standard measures of summary statistics were used to describe the data: relative and absolute frequencies for categorical variables and arithmetic means with standard deviations for continuous variables. The chi-squared distribution test was used to compare fertilization, blastulation, and utilization (yes/no) of oocytes with/without detectable spindles. Evaluation of the association between the presence of the spindle and the formation of blastocyst was the main endpoint of the statistical analysis. For this purpose, generalized estimating equations (GEE) logistic regression with repeated measurements was used. According to the quasi-likelihood under independence model criterion (QIC), an independent correlation structure was chosen. The dependent variable was the formation of the blastocyst (yes/no); independent variables were the presence of a spindle (yes/no), the presence of severe male infertility (yes/no), and the age of female patients. Results with a *p* value < 0.05 were considered statistically significant. Statistical analysis was performed using SAS software version 9.4.

## Results

### PLM assessment prior to scheduled ICSI

A total of 916 PB-displaying oocytes were PLM-examined and classified as A, B, C, or D based on the observed birefringence pattern (Fig. 1a, criteria described in the “Materials and Methods” section). Only 512/916 (55.90%) of analyzed oocytes exhibited PB already at the time of retrieval (MII oocytes), while 404 oocytes (44.10%) extruded PB in vitro shortly after retrieval (MI/MII). The MII spindle (oocyte grade A or B) was detected in 89.84% MII oocytes and 38.86% MI/MII oocytes (Fig. 1b). Together, nearly a third (299/916, 32.64%) of examined oocytes from poor/slow-responding patients showed no MII spindle (grade C or D) at 3–4 h after OPU. This result was in sharp contrast with the data from the maturity assessment of the control group of donor eggs. Oocytes from egg donors typically displayed PB at the time of retrieval and the MII spindle was absent in only 3.22% (13/404) of collected oocytes (Fig. 1b).

If the majority of oocytes within the treatment cycle exhibited an MII spindle, fertilization took place immediately after PLM examination. A total of 682 oocytes (432 MII and 259 MI/MII oocytes) were injected in standard ICSI time—39–40 h after hCG (Supplementary Table 1).

### Delayed ICSI and PLM re-examination

**Fig. 2:**
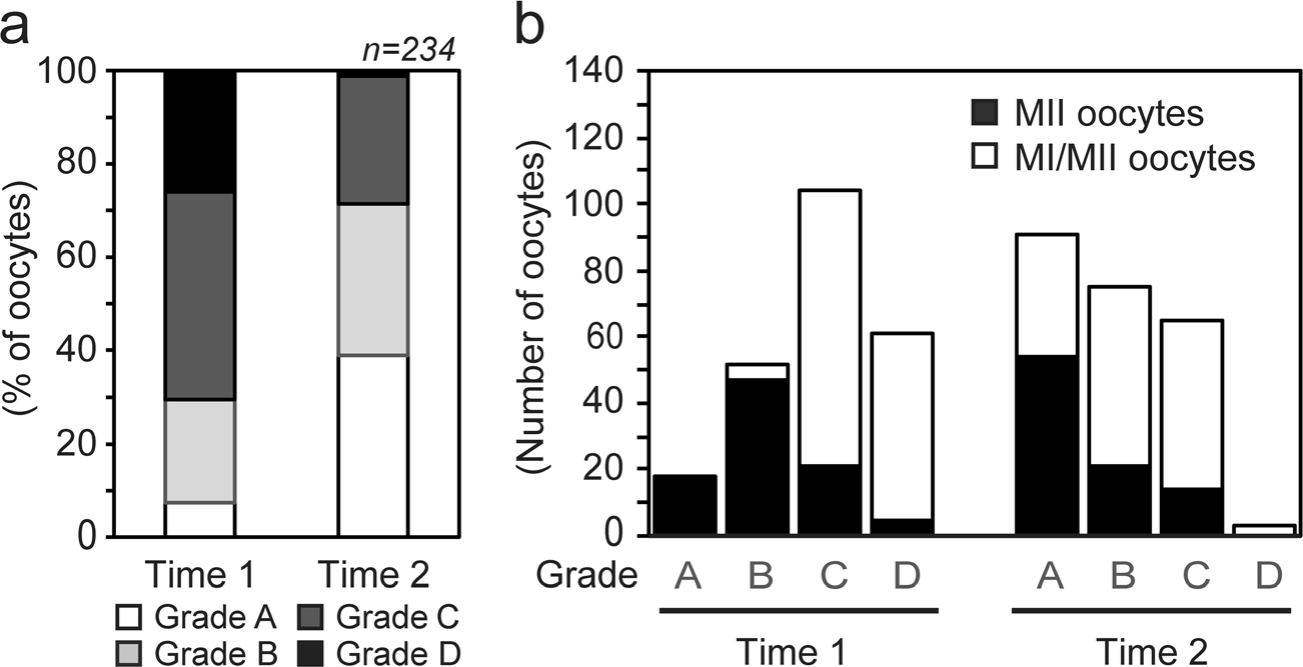
Oocyte grades in cycles with postponed ICSI. (a) The percentage of oocyte grades and (b) number of MII and MI/MII oocytes within oocyte grade categories detected during the initial (time 1) and later (time 2) PLM examination

PLM examination revealed that the absence of a spindle signal was abundant in MI/MII oocytes (Fig. 1b). Thus, we decided to test whether the absence of the MII spindle might be only temporary. We assumed that the spindle signal would develop as late-maturing oocytes progressed in maturation. If the majority of oocytes within the treatment cycle was found to lack MII spindle signal, ICSI was deliberately rescheduled to a later time (Supplementary Table 1). A total of 234 oocytes (89 MII oocytes and 145 MI/MII oocytes) from 86 cycles were not injected immediately after the first PLM assessment (time 1). Instead, the group of oocytes was kept in culture and re-inspected for presence/absence of the MII spindle prior to delayed ICSI ~ 2 h later (time 2). The PLM pattern has changed in 70.51% of analyzed oocytes and the presence of the spindle dramatically increased from 29.49% at time 1 to 71.37% at time 2 (Fig. 2a). The fact that 98/165 (59.39%) of initially spindle-negative oocytes displayed a spindle signal at a later time demonstrated that birefringence pattern is not a fixed quality of the given oocyte but may change over time (Fig. 2a, b). Interestingly, 86.73% of eggs that developed a spindle signal during extended incubation period were MI/MII oocytes (Fig. 2b). Provided with the extra incubation time, C-graded oocytes were more likely to assemble a detectable MII spindle than D-graded oocytes which represents an earlier stage of oocyte development (Fig. 2b). Together, this data indicates that late-maturing oocytes, which failed to complete development in vivo, require extra time to achieve full maturity in vitro.

### Fertilization, blastulation, and utilization

**Fig. 3:**
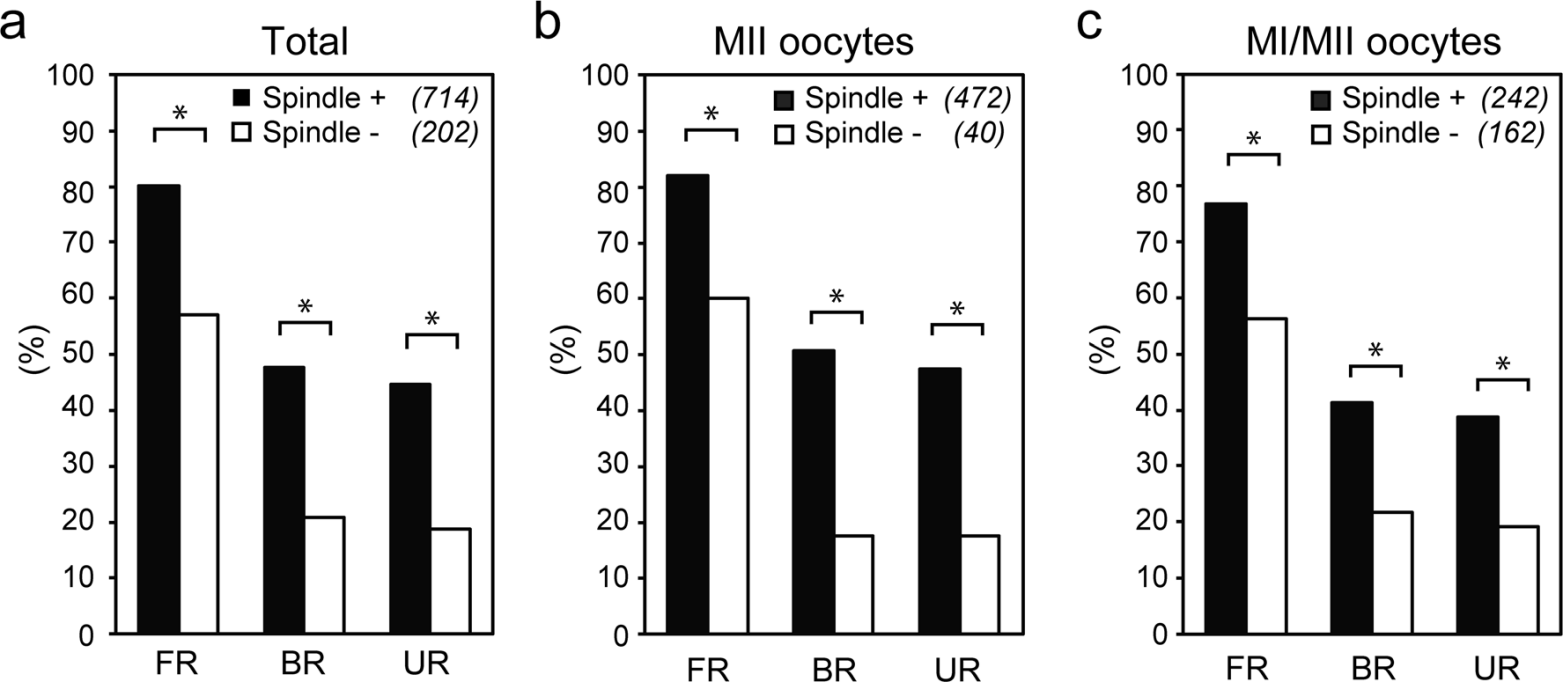
Impact of oocyte grade on fertilization and embryo development. (a) Comparison of fertilization (FR), blastulation (BR), and utilization (UR) rates of oocytes with (+) or without (−) spindle in the total of analyzed oocytes, (b) only MII oocytes, and (c) only MI/MII oocytes. *p ˂ 0.001

Postponing the ICSI in indicated cycles, we increased the overall proportion of oocytes with an MII spindle from 67.36 to 77.95% (Fig. 1b, Supplementary Table 2). As expected, oocytes in which an MII spindle signal was detected before ICSI showed significantly higher developmental competence than oocytes without a spindle (Fig. 3a). A-graded oocytes had higher fertilization, blastulation, and utilization rates than grade B oocytes. Spindle-negative oocytes (grades C and D) were less successful in all analyzed parameters (Supplementary Table 2). The same trend was apparent in subpopulations of MII and MI/MII oocytes (Fig. 3b, c; Supplementary Table 2). The MI/MII oocytes showed generally lower developmental potential than the MII oocytes. However, the rate of fertilization and embryonic development was acceptable if they managed to assemble a spindle prior to ICSI (Fig. 3c, Supplementary Table 2).

Importantly, the results of logistic regression proved that there is an association between the presence of the spindle and the formation of the blastocyst. According to the odds ratio, oocytes with an MII spindle signal have 3.4 times higher probability of formation of blastocyst than oocytes without a detectable MII spindle. The presence of the spindle is also the only statistically significant variable (*p* < 0.001) in multivariate analysis. Confounding factors, namely severe male infertility and female age, turned out not to be statistically significant (Table 1). Logistic regression using different oocyte grades (A, B, C, or D) as a potential predictor of formation of blastocyst was performed as well, choosing type A as a reference type. Odds ratios show that blastocysts have a significantly (*p* < 0.001) lower probability of formation if they originate from oocytes graded B, C, and D (odds ratios 0.492, 0.216, and 0.113, respectively) instead of A-graded oocytes (Table 1). For other variables, results are very similar to previous regression, and male infertility factor and female age remain statistically insignificant.

**Table 1:** GEE logistic regression evaluation. Evaluation of the association between blastocyst formation and presence/absence of the spindle or blastocyst formation and oocyte grades (A-D)

Parameter	OR (95% CI)	*p* value
Presence of the spindle	3.439 (2.301, 5.140)	< 0.001
Male infertility factor	0.767 (0.469, 1.253)	0.2890
Female age	0.974 (0.937, 1.011)	0.1652
Grade A	1 (reference)	
Grade B	0.492 (0.358, 0.676)	< 0.001
Grade C	0.216 (0.135, 0.345)	< 0.001
Grade D	0.113 (0.042, 0.301)	< 0.001
Male infertility factor	0.743 (0.449, 1.230)	0.2483
Female age	0.974 (0.939, 1.011)	0.1626

*OR* odds ratio, *CI* confidence interval

### Clinical outcome

A total of 197 transfers of 216 embryos were carried out. A vast majority (90.74%) of the transferred embryos were derived from oocytes which developed a detectable spindle (grade A or B) prior to ICSI (Supplementary Table 3). Implantation of A-graded oocytes was slightly higher than B-graded oocytes (PR 48.15% vs. 42.68%) but their chance of producing clinical pregnancies was comparable (CPR 35.80% vs. 36.59, respectively). Embryos derived from grade B oocytes showed a higher risk of abortion at later stages of pregnancy (Supplementary Table 3). Only 3/15 (20%) of individually transferred embryos derived from oocytes without a spindle produced clinical pregnancies. Only two embryos derived from D-graded oocytes were transferred along with their sibling embryos from spindled oocytes. Interestingly, the combination of embryos derived from spindle-positive and spindle-negative oocyte always resulted in singleton pregnancies. Twin pregnancies were recognized only in two patients who had two embryos derived from A-graded oocytes transferred. In summary, 47/49 live births originate from transfers involving at least one embryo derived from the spindle-positive oocyte (Supplementary Table 3). Further 8 ongoing clinical pregnancies are still being followed, 7 of them are produced by spindled oocytes. Notably, 28.24% of transferred embryos were derived from MI/MII oocytes and 11/51 healthy children reported so far by study participants were born from these late-maturing oocytes extruding PB in vitro (Supplementary Table 1).

## Discussion

In this study, we explored the clinical value of spindle imaging in a group of patients with unexpectedly suboptimal response to ovarian stimulation. We used PLM to inspect whether oocyte completed nuclear maturation prior to ICSI. Non-invasive monitoring of microtubular dynamics during MI to MII transition assisted us to time ICSI according to the developmental stage of the late-maturing oocytes. Combining “rescue in vitro maturation” of MI oocytes with PLM-navigated optimization of timing of ICSI, we were able to effectively utilize oocytes extruding PB in vitro.

Immature oocytes retrieved in stimulation cycles are generally regarded as of poor quality and rejected for fertility treatment because of the risk of abnormal fertilization [30, 31]. Nevertheless, published case reports indicate that immature oocytes can acquire developmental competence during overnight culture [6–10]. Here, we focused on clinical utilization of developmentally delayed oocytes that extruded PB in vitro shortly after retrieval. Our data demonstrated that these MI/MII oocytes can produce viable embryos and full-term pregnancies if the time of ICSI is adjusted to their maturational stage. Hence, poor developmental outcomes of late-maturing oocytes reported in previous studies might be, at least partially, attributed to untimely sperm injection [2–5].

Although being of paramount clinical importance, the timing of fertilization remains a subject of controversy. Clinical data support the notion that a pre-incubation interval between oocyte retrieval and ICSI improves the outcome of IVF treatment [12, 14, 32, 33]. However, other studies recommended limiting the cultivation period to prevent post-ovulatory aging of the oocyte [34, 35]. In this study, ICSI was performed on the day of retrieval and total pre-incubation time never exceeded 9 h, the period associated with a decline of post-fertilization outcome [5, 32]. Oocyte denudation was carried out immediately after retrieval, so we could discriminate between (1) in vivo matured MII oocytes and (2) MI/MII oocytes which extruded PB in vitro. Our results confirmed previous findings that these two subpopulations differ in incidence of the MII spindle and their developmental potential [28]. If denudation was performed just before ICSI, MII and MI/MII oocytes would not be distinguishable based on morphology. Confocal microscopy clearly demonstrates that the oocyte appearance in transmitted light can be misleading. Some seemingly mature, PB-displaying oocytes might be engaged in chromosome segregation or early phases of spindle reconstitution (interkinesis), and are thus not ready for fertilization. Polarized light microscopy can serve as a tool to discriminate oocytes arrested in an MII stage from those undergoing the maturational transition from the MI to MII stage (Supplementary Figure).

It has been previously suggested that non-invasive spindle imaging might be used for egg quality control, and to optimize the timing of ICSI in clinical practice [4, 11, 17–19]. Strikingly, reported incidence of spindle-negative oocytes varied from 12 to 56% [11, 17–28]. One of the reasons for this inconsistency could be that the authors worked with different sources of oocytes. In this study, nearly one-third of oocytes from poor/slow responders lacked a detectable MII spindle. This was in contrast to control oocytes from egg donors which typically showed a spindle signal. Also, the representation of late-maturing MI/MII oocytes in an analyzed sample has not been taken into account in most studies. Our data show that not only a maternal age [18, 26, 36] and infertility factor [37], but also the suboptimal response to the ovarian stimulation and the timing of observation affect the incidence of spindle-positive oocytes in an analyzed population. In addition, procedural diversities and different laboratory conditions could have influenced PLM examination results. In this study, a great deal of effort was dedicated to optimizing the spindle imaging procedure and minimizing factors known to adversely affect the stability of delicate division machinery, namely pH and temperature fluctuation [3].

Published studies also differ in the classification of the spindle pattern, study endpoints, and interpretation of results. Some studies only compared oocyte as with and without a spindle [11, 17–20], and others recognized dysmorphic and translucent spindle morphology [21–23]. Here, we classified oocytes into four categories (A–D) based on the observed birefringence pattern. We distinguished between bipolar (grade A) and dysmorphic/translucent spindles (grade B), as well as the absence of spindle birefringence (grade C) and presence of microtubule bridge (grade D). When interpreting a detected spindle pattern, we took advantage of the knowledge of spindle dynamics during oocyte maturation [1, 2]. Our presumption was that, at least in some oocytes observed shortly after PB extrusion, the absence of spindle birefringence might be only temporary. Indeed, more than half of spindle-negative oocytes managed to form a detectable MII spindle when ICSI was postponed to a later time. The delay in spindle signal emergence is explained by the fact that, in the absence of centrosome, microtubule nucleation is slow and it may take a couple of hours for a bipolar MII spindle to be reformed after PB extrusion [1, 2]. Capability to assemble the MII spindle is likely to reflect overall oocyte fitness because spindle-positive oocytes have been shown to contain more mtDNA copies and ATP content than oocytes without a spindle [38].

Multifactorial constitution of egg quality makes it difficult to evaluate in clinical practice. Nevertheless, this study confirmed that the presence of the spindle is significantly associated with the capacity of the oocyte to form a blastocyst. Late-maturing MI/MII oocytes have generally lower developmental competence than in vivo matured MII oocytes. However, the rate of embryo development was acceptable if they managed to assemble a detectable spindle prior to ICSI. Importantly, 10 out of 42 children born in this study were conceived by individually timed fertilization of late-maturing MI/MII oocytes which are typically discarded. We have shown that the “rescue in vitro maturation” of immature oocytes can be used as a salvaging method and viable option to the cancelation of the IVF cycle if there are no MII oocytes available for ICSI.

## Conclusions

This study demonstrates the benefits of spindle imaging in IVF cycles with a suboptimal response to conventional stimulation. In particular, we used PLM to determine the maturation status of late-maturing oocytes and, when required, postponed ICSI so that the oocytes could complete maturation in vitro. The timing of sperm injection needs to be individually fine-tuned especially for MI oocytes which extrude PB in vitro, and are thus at risk of premature fertilization. Therefore, we suggest the employment of egg maturity assessment in indicated patients with a low number of MII oocytes available for ICSI. Based on our experience, a subtle change in laboratory procedures, namely individual adjustment of the timing of ICSI, can make a major difference for poor prognosis patients.

## Electronic supplementary material

Supplementary Figure Correlation of chromosome-microtubule organization with birefringence pattern in oocytes representing stages of MI to MII transition during oocyte maturation. The appearance of oocytes in transmitted light (top row), combined with a fluorescent signal for chromosomes (cyan) and microtubules (magenta) (middle row), and in polarized light (bottom row) are shown. Each oocyte was first PLM-examined and immediately fixed. Fixed oocytes were (immuno) labeled with Hoechst (DNA) and anti-α-tubulin antibody (microtubules). Fluorescent and transmitted light images were acqured by Zeiss LSM 800 confocal laser scanning microscope. Scale bar, 20 μm. The yellow arrow indicates the presence of PB and the white arrow highlights the position of birefringent microtubules. In vitro matured, supernumerary immature oocytes donated for research were used for this experiment. (PNG 2828 kb)

High resolution image (TIF 32126 kb)

Supplementary Table 1 Overview of standard/delayed ICSI outcomes in MII, MI/MII, and the total of oocytes. (XLSX 11 kb)

Supplementary Table 2 Impact of oocyte grade on fertilization, embryo development, and clinical outcome. Overview of the total of analyzed oocytes and subpopulations of MII and MII oocytes. (XLSX 12 kb)

Supplementary Table 3 Oocyte grades and clinical outcome. Overview of clinical outcomes of single/double embryo transfers with respect to oocyte grade. (XLSX 10 kb)
