# Supplementary figures and images for: Egg maturity assessment prior to ICSI prevents premature fertilization of late-maturing oocytes
Source: J Assist Reprod Genet. 2019 Jan 12;36(3):445–52. doi: 10.1007/s10815-018-1393-0 (PMC6439061; doi:10.1007/s10815-018-1393-0)

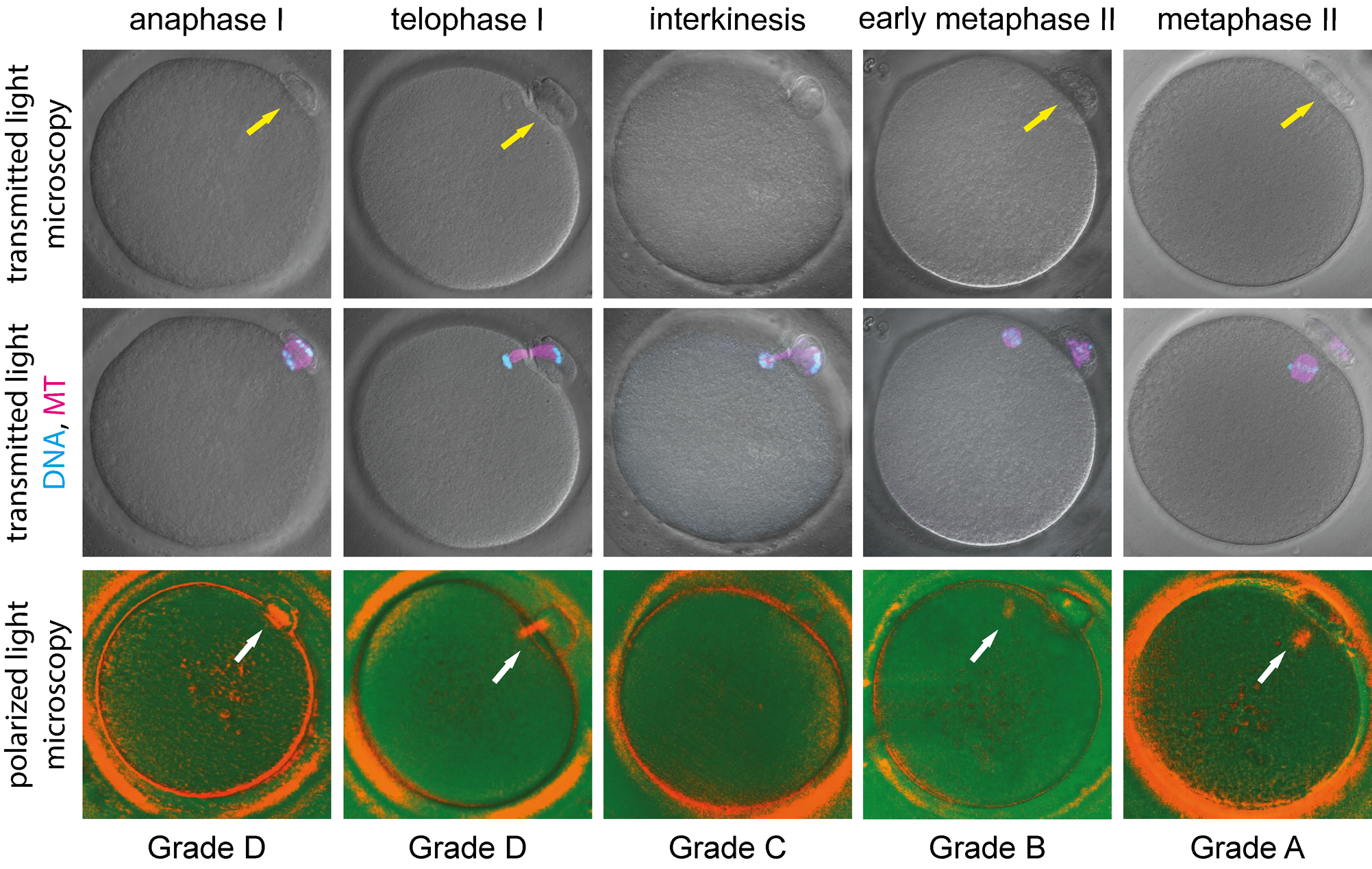

Supplement: Supplementary file 1 — Correlation of chromosome-microtubule organization with birefringence pattern in oocytes representing stages of MI to MII transition during oocyte maturation. The appearance of oocytes in transmitted light (top row), combined with a fluorescent signal for chromosomes (cyan) and microtubules (magenta) (middle row), and in polarized light (bottom row) are shown. Each oocyte was first PLM-examined and immediately fixed. Fixed oocytes were (immuno) labeled with Hoechst (DNA) and anti-α-tubulin antibody (microtubules). Fluorescent and transmitted light images were acqured by Zeiss LSM 800 confocal laser scanning microscope. Scale bar, 20 μm. The yellow arrow indicates the presence of PB and the white arrow highlights the position of birefringent microtubules. In vitro matured, supernumerary immature oocytes donated for research were used for this experiment. (PNG 2828 kb) [file 10815_2018_1393_Fig4_ESM.png]

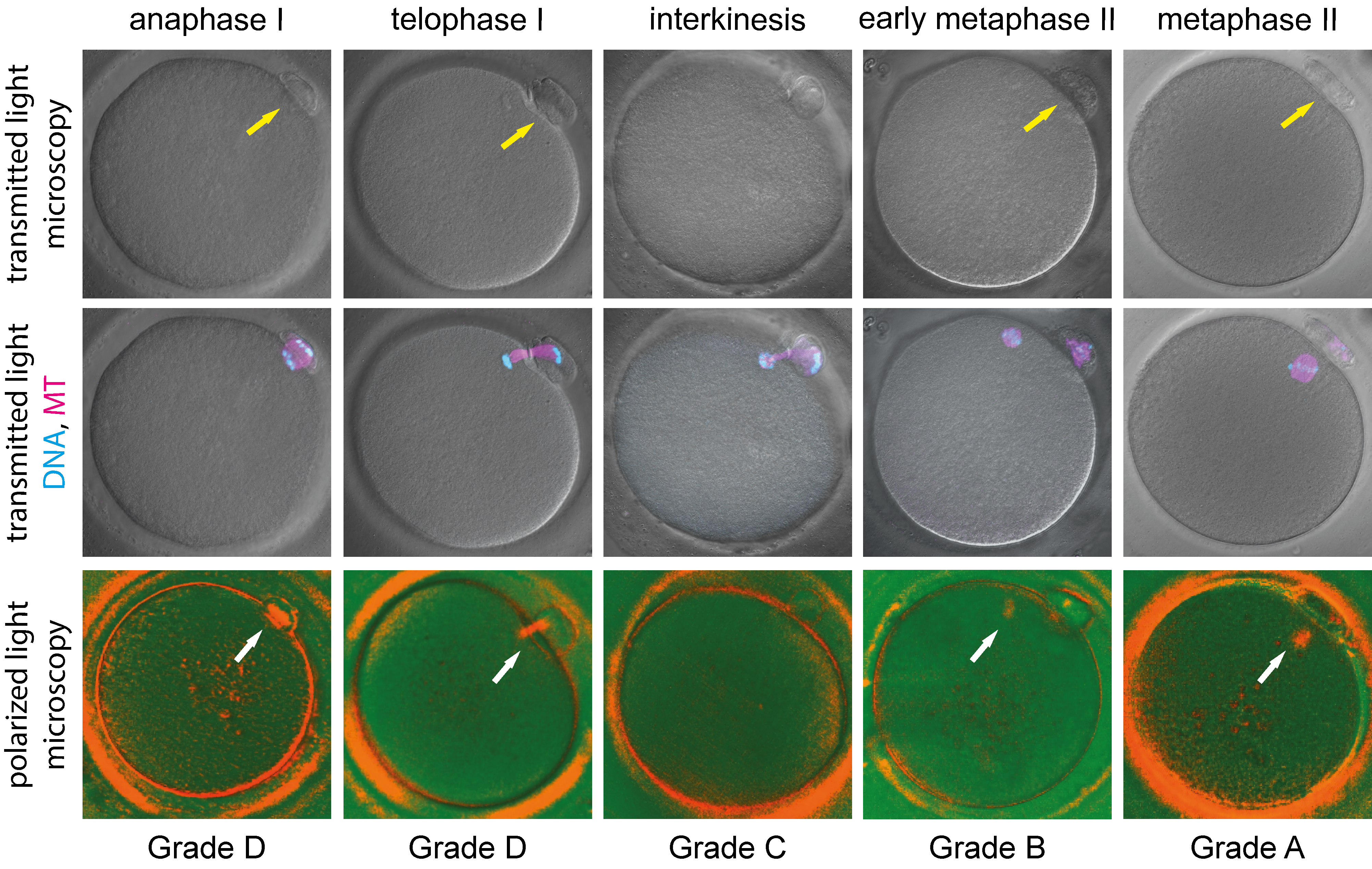

Supplement: Supplementary file 2 — High resolution image (TIF 32126 kb) [file 10815_2018_1393_MOESM1_ESM.tif]
